# Geriatric depression and quality of life in North Shoa Zone, Oromia region: a community cross-sectional study

**DOI:** 10.1186/s12991-021-00357-z

**Published:** 2021-07-28

**Authors:** Kemal Jemal, Dejene Hailu, Bikila Tesfa, Tasfaye Lama, Tadele Kinati, Endeshaw Mengistu

**Affiliations:** 1 Department of Nursing, College of Health Sciences, Salale University, Fitche, Ethiopia; 2 Department of Psychology, College of Social Sciences and Humanities, Salale University, Fitche, Ethiopia

**Keywords:** Geriatric depression, Quality of life, Associated factors, North Shoa Zone

## Abstract

**Introduction:**

Depression and low quality of life are severe conditions that lead to disability and mortality, common in high and low-resourced countries. Therefore, this study aimed to assess geriatric depression, quality of life, and associated factors among elderly persons in the low-resource country.

**Methods:**

A community-based cross-sectional study was used from March to April 2020. The depression and quality of life were assessed using the standardized and pre-tested geriatric depression scale (GDS) and the World Health Organization Quality Of Life (WHOQOL)-BREF, respectively. A multi-stage sampling technique was employed to select woreda and study participants. For data input and analysis, Epi-data version 4.3 and SPSS version 23 were utilized, consecutively. Bivariable and multivariable in the logistic regression analysis were done, and significance was determined at the odds ratio with a 95% confidence interval and *P* value < 0.05.

**Results:**

A total of 822 elderly persons participated in face to face interviewed-administered questionnaire. More than half 54.5% (*n* = 448) of elderly persons had depression and 51.8% (*n* = 426) of elderly participants had low quality of life. Advanced age, single participants, not having a formal education, living alone, and having chronic diseases were significantly associated with both the depression symptoms and low overall WHOQOL-BREF. Depression was correlated with low quality of life.

**Conclusion:**

We found that elderly persons have a high risk of depression symptoms and a low quality of life. The Ethiopian Ministry of Health should develop psychological interventions, providing ongoing education for the elderly, and monitoring the health of the elderly population to address the specific needs of elderly persons who have been impacted by the aging process.

## Background

Depression and low quality of life are common problems that accelerating the risk of morbidity and mortality among elderly persons [1, 2]. Worldwide, people live longer; more than 125 million people are aged 80 years and older [3]. By 2050, there will be almost 434 million elderly persons worldwide, of which 80% of all elderly people will live in low- and middle-income countries, with an estimated 90% of health expenditure was needed for home care services [3, 4].

Aging is the time of life when emotional weakness is observed [5]. It is a period of numerous illnesses that emerged and is vulnerable to certain diseases, disorders, poor quality of life, and accidents repeatedly [6]. Additional to neurobiological changes in the brain, aging predictably involves a significant loss over the years, not only in terms of individuals’ emotions but also in terms of their physical condition and social status [7].

Depression significantly affects the elderly over the age of 60 years [8]. It magnifies the disabilities of a person through physical illness, failure to treatment and rehabilitation, and decline in an individual cognitive function [9, 10]. Because of numerous reasons such as lifespan, instability, the development of chronic degenerative illnesses, impairment of autonomy, and poor family structures, the elderly quality of life is affected [11]. A meta-analysis study over 75-year-olds revealed that the point prevalence of major depression was 7.2%, with women’s rates ranging from 4.0 to 10.3% and men’s rates ranging from 2.8 to 6.9% [12].

The prevalence of depressive symptoms in the elderly population was 54.4% in Taiwan [13], 40.6%) in Pakistan [14], and 27.8% in South Korean [15]. In India, old age at home was 85.7% with a low quality of life, while 63.6% of individuals have an average quality of life [16]. Another community study done in India found that depression symptoms were 58% and 28% of elderly persons had a low quality of life [17]. Community studies in South Africa reported that the prevalence of depressive symptoms in older adults was ranged from 40 to 50.1% [18, 19]. In Ethiopia, a community study done in Ambo town showed that the prevalence of geriatric depression was 41.8% [20].

Depression in the elderly causes a significant loss of skills, hastens the progress of a physical disease, enhances deaths due to suicide and physical diseases, and high consumption of healthcare services [15, 21]. Similarly, it leads to loosed of social harmony, poor self-care management, deteriorated quality of life, and multiple episodes of diseases [22, 23].

The way an elderly person lives has a direct impact on his or her aging process, socioeconomic and biological risks are affecting his/her health and quality of life in the community [24]. The quality life of the elderly is affected by advanced age, low income, retirement, chronic disease, physiologic change of body function, loss of social support, and loneliness [6, 15]. This leads to a mental health problem, disability, and multi-morbidity, in which one-seventh of elderly persons have depressive symptoms and low quality of life [25, 26].

Improving the quality of life and symptoms of depression may reduce the burden on family and healthcare providers [19]. Alongside, addressing physical and psychological health problems and physiological changes may be vital for improving elderly mental health and quality of life [27, 28]. Early identifying and treating the elderly symptom of depression and improving the elderly quality of life is essential to keep the elderly on the wellness track [29, 30].

As far as we know, there has been limited evidence-based literature concerning geriatric depression and quality of life in the elderly population in low-resource countries, particularly in Ethiopia. Hence, this study provides key findings for the geriatric population, which supports the development of nationwide intervention of the elderly persons’ mental well-being. Therefore, we aimed to assess geriatric depression, quality of life, and associated factors among elderly persons.

## Methods

### Study design, area, and period

A community-based cross-sectional study was employed from March to April 2020 in the North Shoa Zone of Oromia Regional State. The zone’s capital city is Fitche, located at a distance of 112 km from Addis Ababa in the north direction. North Shoa Zone has a total population of more than 1.6 million, where 820,595 are male and 818,992 are female. Eighty-eight percent (1,447,330) of the North Shoa population lives in rural areas, and 12% (192,105) live in urban areas. North Shoa has contained a higher number of elderly due to the presence of Debra Libanos *Gadam* (*monastery*), which consists of more than one-fourth (26.3%) of the district population are aged above 60 years old. The North Shoa Zone has two general hospitals and two district hospitals, 63 primary health centers, and 268 health posts.

### Study population

The definition of old age depends on various countries’ settings to determine the old age cutoff point. The United Nations uses 60 years to define old age and recommends the age range of 50–65 years to be used as a cutoff point by countries [31]. In Ethiopia, the cut points of old age are started from 60 years [32]. Therefore, our study participants were all selected elderly population whose age greater than or equal to 60 years and who have been living in the North Shoa Zone for more than 1 year were included in the study. Those who are critically ill, have hearing problems, and have a history of dementia were excluded from the study.

### Sample size determination and sampling technique

The sample size required for this study was calculated using the formula used to determine a single population proportion. We considered a proportion of 41.8% from the study done in Ambo town on geriatric depression [20] and a 95% confidence interval with a 5% margin of error. Using the design effect of two, the final sample size was 822.

A multi-stage sampling technique was used to select study participants with the assumption of a homogenous population. North Shoa has 14 districts; 6 districts (Aleltu, Debra Libanos, Degam, Hidabo Abote, Wechale, and Yaya Gulele) were selected by the lottery method. One Kebele (the least local administration) was selected and clustered into six *goxs* (clusters) with a total of 36 goxs/clusters for all kebele from each district. Each cluster list of the household folder was obtained from health extension workers, and then a list of the household was prepared. After that, 40% of the households were selected by computer-generated random sampling technique from the household list. Finally, every household in the selected clusters was visited for an interview.

### Study variables

The outcome variables for the study were depression and quality of life. Depression was measured with a GDS that contains 15 items of questions. The question is self-reporting on how the person felt during the last week of his/her life. The study participants were asked to answer the questions as “yes” or “no”. Each bold answer indicated depression (‘yes’ or ‘no’) counts one point and zeros the other. The sum is accepted as a total depression score with a cutoff point of greater than or equal to five. The scores to be obtained from the scale are between 0 and 15. The outcome of depression was coded depending on the cutoff point 0–4 are considered normal, 5–8 revealed mild depression, 9–11 indicate moderate depression, and 12–15 indicated severe depression [33]. The GDS was validated in other country among older adults were satisfactory with (Cronbach *α* = 0.80) and test–retest reliability (*r* = 0.73) [34]. Even though the GDS was not validated in Ethiopia, several studies were conducted using translated GDS into Afan Oromo and Amharic languages [20, 35]. In addition, we checked the suitability of GDS in the pre-test of this study with a Cronbach-alpha value of 0.81.

The other outcome variable is quality of life, which was measured with the WHOQOL-BREF. It contains 26 questions categorized into four sub-domains that are scored on a 5-point Likert scale. The lowest score obtained from the scale is 24, the highest is 120, and two questions were measured overall health-related quality of life and general health perception. The sub-domains were included the physical domain, psychological domain, social domain, and environmental domain. The mean score of items within each domain is used to calculate the domain score. Mean scores are then multiplied by 4 to make domain scores comparable with the scores used in the WHOQOL-100. An overall WHOQOL-BREF score can be calculated by summing up each singular score value of the mean. Higher scores or above mean indicated a better quality of life and below the mean low quality of life [36]. The outcome of WHOQOL-BREF was calculated after testing normality distribution, taking the mean scores as a cutoff point that was 68.34. Then ≥ 68.34 is determined the high quality of life and below the mean was indicted low quality of life. The WHOQOL-BREF was validated in Ethiopia, the reliability of each domain found that Cronbach’s alpha value of 0.84 for the physical domain, 0.74 for the psychological domain, 0.58 for the social domain, and 0.71 for the environmental domain [37]. It was also reliable in our pre-tested study with Cronbach’s alpha value of 0.78 for the physical domain, 0.83 for the psychological domain, 0.69 for the social domain, and 0.75 for the environmental domain.

### Independent variables

The included independent variables were socio-demographic characteristics [age, gender, marital status, education, number of children, place of residence and living status (alone, with a married child, with spouse, with spouse and child)], source of income [retirement, helped by family, farmers, merchant and no income/non-governmental organization (NGO) support], social support, chronic disease, and substance use. Social support was measure using Oslo-3 social support tools with scores of 3–8 was categorized as poor social, and above 9 was strong social support [38]. In our study, Oslo-3 social support was reliable in the pre-tested sample of participants with Cronbach-alpha = 0.79. Current substance use was defined as using at least one of the specified substances (alcohol, chewing khat, tobacco, and coffee/caffeine) in the past 3 months. Chronic disease was asked if the older adults have one or more of the following diseases for more than 6 months (HIV/AIDS, tuberculosis, cardiovascular disease, cancer, diabetes mellitus, chronic respiratory disease, and chronic musculoskeletal diseases).

### Data collection tools and procedure

The data were collected using standardized and pre-tested GDS [33], and the WHOQOL-BREF [36] was used to assess the level of depression and quality of life, respectively. Ten BSc nurses were recruited for data collection and five supervisors for supervision. The questionnaire contains different components: socio-demographic, living status, source of income, social support, co-morbidity, substance use, depression, and quality of life.

Data collectors and supervisors were trained for 5 days on the study’s purpose, details of the questionnaire, interviewing techniques, the importance of privacy, and ensuring the respondents’ confidentiality. In addition to data collection training, data collectors and supervisors have trained the prevention technique of the COVID-19 pandemic and provided personal protective equipment.

The questionnaire was prepared in English language and translated to Afan Oromo and Amharic languages and back-translated to the English language-by-language experts to check its consistency. The pre-test was done on 5% (41 samples) in Girar Jarso *district* other than the study area having the same socio-demographic characteristics. Close supervision at the end of every data collection was made; the questionnaire was reviewed and checked for completeness, accuracy, and consistency by the supervisor and principal investigator to take timely corrective measures before actual data collection.

### Data processing and analysis

Data were coded, edited, cleaned, and entered into Epi-data and transported to SPSS version 23. The descriptive data analysis was done and presented in frequency, summary statistics, table, and graphs. For binary logistic regression, the cutoff point of ≥ 5 was used for depressed, coded as “1”, and < 5 was normal, coded as “0”, and the mean was also used for quality of life, coded “1” for the low quality of life (below the mean) and “0” for the high quality of life (above the mean). The analytic part was analyzed, odds ratio with a 95% confidence interval, and a two-tailed *P* value was calculated to identify the presence and strength of association. Variables with *P* value ≤ 0.2 in the binary analysis were included in a multivariable logistic regression analysis to control the confounding effect variables [39]. The model fitness was checked using the Hosmer and Lemeshow goodness-of-fit model and the model was fitted at *P* value = 0.628 for depression and 0.574 for quality of life. Statistical significance was declared if *P* value < 0.05.

### Ethical consideration

Ethical clearance was obtained from the Salale University Ethical Review Committee. All methods were performed in accordance with the relevant guidelines and regulations. Written consent was sought from the North Shoa Zonal health bureau/woreda department. The informed written consent form was obtained from each respondent. The respondents were informed that their inclusion in the study is voluntary, and they are free to withdraw from the study if they are not willing to participate. If any question they do not want to answer, they have the right to do so. The confidentiality of respondents was ensured by excluding their names from the questionnaire and kept in a password-locked computer.

## Results

A total of 822 participants have participated with zero none-response rates. Participants’ mean age was 75.46 (± standard deviation = 8.80), with the age range from 60 to 120 years. Greater than half (51.3%) of study participants were male, and 309 (37.6%) were married. The majority (70%) of the respondents had no formal education, and 394 (47.9%) had more than three children. Three-seventh of 354 (43.1%) of the study participants had no source of income. The majority (71.8%) of the study participants were from rural areas, and 82.2% of the elderly were traveled less than or equal to 5 km to access health care facilities. Two-thirds (66.9%) of the elderly had no social support, and 380 (46.2%) lived alone. Greater than one-fourth (26.0%) of the respondents have chronic diseases, and only 330 (40.2%) of the study participants had no current substance use (Table 1).

**Table 1:** Socio-demographic and economic characteristics among the elderly at North Shoa Zone, Oromia region, Ethiopia, 2020 (*n* = 822)

Variables	Number of respondents	Percent
Age		
60–69	265	32.2
70–79	303	36.9
80–89	198	24.1
≥ 90	56	6.8
Sex		
Male	422	51.3
Female	400	48.7
Marital status		
Single	164	20.0
Married	309	37.6
Widowed	228	27.7
Divorced	121	14.7
Educational status		
No formal education	578	70.3
Literate/formal education	244	29.7
Number of children		
No children	190	23.2
One child	80	9.7
Two children	158	19.2
Three and above	394	47.9
Source of income		
Retirement	34	4.1
Helped by family	148	18.0
Farmer	244	29.7
Merchant	42	5.1
No income	354	43.1
Place of residence		
Urban	232	28.2
Rural	590	71.8
Traveling the distance to receive health care		
≤ 5 km	676	82.2
> 5 km	146	17.8
Living status		
Alone	380	46.2
With a married child	256	31.2
With spouse	94	11.4
With spouse and child	92	11.2
Social support environment available		
No	550	66.9
Yes	272	33.1
Co-morbidity/chronic disease		
No	608	74.0
Yes	214	26.0
Current substance use		
Alcohol	318	38.7
Chewing khat	6	0.7
Tobacco	42	5.1
Coffee/caffeine	126	15.3
No current substance use	330	40.2

### The prevalence of geriatric depression and quality of life

From 822 study participants, 45.5% (*n* = 374) of elderly persons had normal (free from symptoms of depression), 23.1% (*n* = 190) of elderly persons had mild symptoms of depression, 20.0% (*n* = 164) of elderly persons had moderate symptoms of depression and 11.4% (*n* = 94) of elderly persons had severe symptoms of depression.

More than half of the elderly persons were rated as having a good quality of life for WHOQOL-BREF sub-domains that indicated in physical sub-domains (50.60%), psychological sub-domain (56.20%), and social domains sub-domain (51.10%). Conversely, elderly persons had scored 42.30% for the environmental sub-domain. The overall quality of life was calculated using each mean of the WHOQOL-BREF sub-domain. Among the study participants, the proportion of high quality of life for elderly persons who scored ≥ 68.34 mean were 48.2% (*n* = 396), and 51.8% (*n* = 426) of elderly persons had scored below the mean that indicates the low quality of life.

**Fig. 1:**
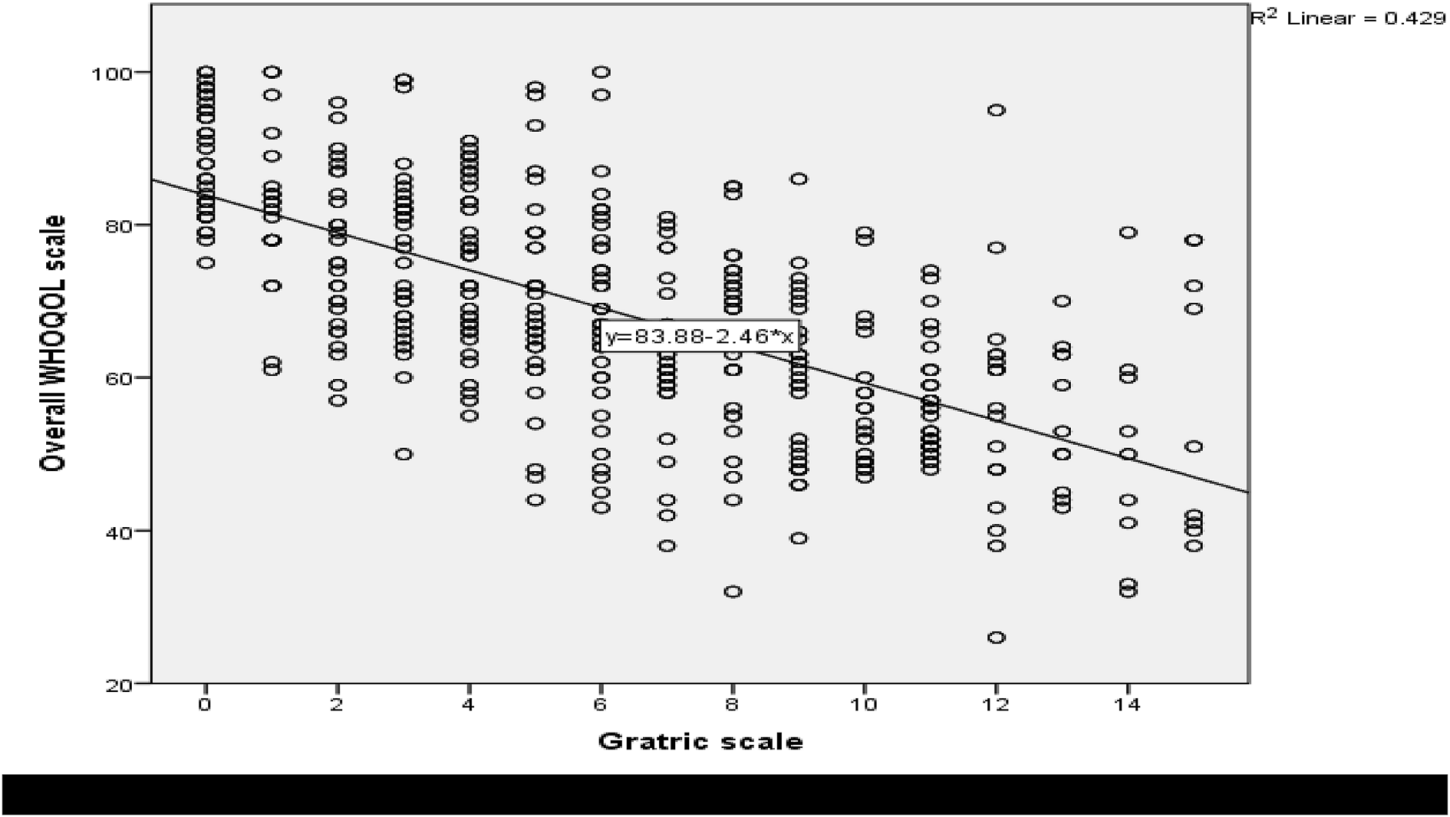
Correlation between depression and overall WHOQOL among elderly persons at North Shoa Zone, Oromia region, Ethiopia, 2020 (*n* = 822)

Figure 1 indicates the correlation of geriatric depression with quality of life. Depression was correlated with quality of life at − 0.655 Pearson Correlation Coefficient. The graph was negatively correlated, as the geriatric depression scale increase the overall WHOQOL-BREF scale decreased. This indicated that depression was correlated with low overall WHOQOL-BREF (Fig. 1).

Tables 2 and 3 documented the multivariable logistic regression associated with depression and low quality of life among study participants. The more age increased, the odds of geriatric depression increased with age compared to the younger age. Single participants [AOR = 1.67; 95% CI (1.12, 2.50)] and not having formal education [AOR = 1.79; 95% CI (1.06, 2.20)] was two times more likely to have depression than their counterparts. The elderly participants who live alone were 1.58 times more likely to have depression than those who live with family [AOR = 1.58; 95% CI (1.05, 2.26)]. The elderly participants who have chronic diseases were twice more likely to have depression when compared to not have chronic diseases [AOR = 2.27; 95% CI (1.50, 3.44)] and lack of social support also one of the predictors for depression by 55% (Table 2).

**Table 2:** Factors (crude and adjusted odds ratios, confidence intervals, and *p* value) associated with geriatric depression among elderly at North Shoa Zone, Oromia region, Ethiopia, 2020 (*n* = 822)

Variables	*Depression*		Crude OR (95% CI)	*P* value	Adjusted OR (95% CI)	*P* value
	No	Yes				
Age						
60–69	160	105	1		1	
70–79	149	154	1.58 (1.13,2.19)	0.001*	1.30 (0.89,1.90)	0.172
80–89	53	145	4.17 (2.80,6.22)	0.001*	3.22 (2.05,5.03)	0.001*
≥ 90	12	44	5.59 (2.82,11.07)	0.001*	3.62 (1.69,7.75)	0.001*
Sex						
Male	194	228	1		1	
Female	180	220	1.04 (0.78,1.37)	0.187	1.38 (0.98,1.93)	0.063
Marital status						
Single	171	342	3.83 (2.84,5.16)	0.001*	1.67 (1.12,2.50)	0.012*
Married	203	106	1		1	
Educational status						
No formal education	234	344	1.98 (1.46,2.68)	0.001*	1.79 (1.06,2.20)	0.042*
Formal education	140	104	1		1	
Number of children						
No children	68	190	2.01 (1.41,2.87)	0.001*	1.00 (0.50,2.00)	0.990
One child	26	54	2.32 (1.40,1.93)	0.001*	1.62 (0.86,3.04)	0.137
Two children	72	86	1.34 (0.92,1.93)	0.268	1.02 (0.64,1.63)	0.921
Three and above children	208	186	1		1	
Source of income						
Retirement	16	18	1		1	
Helped by family	44	104	2.10 (0.98,4.49)	0.056	1.39 (0.57,3.40)	0.465
Farmers	162	82	0.45 (0.22,0.93)	0.031*	0.52 (0.22,1.23)	0.136
Merchant	34	8	0.21 (0.08,0.58)	0.003*	0.32 (0.10,1.00)	0.050
No income/NGO support	118	236	1.78 (0.88,3.61)	0.112	1.48 (0.67,3.30)	0.334
Living status						
Alone	128	252	2.47 (1.86,3.28)	0.001*	1.58 (1.05,2.26)	0.035*
With family/relatives	246	196	1		1	
Social support						
No	298	252	0.33 (0.24,0.45)	0.001*	0.55 (0.38,0.80)	0.002*
Yes	76	196	1		1	
Chronic illness						
No	308	300	1		1	
Yes	66	148	2.30 (1.65,3.21)	0.001*	2.27 (1.50,3.44)	0.001*
Current substance use						
No	108	222	1		1	
Yes	266	226	0.41 (0.31,0.55)	0.001*	0.70 (0.48,1.02)	0.060

**Table 3:** Factors (crude and adjusted odds ratios, confidence intervals, and *p* value) associated with low OWHOQOL among elderly at North Shoa Zone, Oromia region, Ethiopia, 2020 (*n* = 822)

Variables	OWHOQOL		Crude OR (95% CI)	*P* value	Adjusted OR (95% CI)	*P* value
	High	Low				
Age			17,270/22630			
60–69	155	110	1		1	
70–79	157	146	1.31 (0.94,1.83)	0.111	1.05 (0.69,1.49)	0.943
80–89	68	130	2.69 (1.84,3.95)	0.001*	0.31 (0.34,0.82)	0.004*
≥ 90	16	40	3.52 (1.88,6.61)	0.001*	0.25 (0.22,0.96)	0.038*
Sex						
Male	212	210	1		1	
Female	184	216	1.19 (0.90,1.56)	0.224	1.23 (0.88,1.72)	0.218
Marital status						
Single	177	336	4.62 (3.40,6.27)	0.001*	1.95 (1.53,3.86)	0.001*
Married	219	90	1		1	
Educational status						
No formal education	234	344	2.90 (2.12,3.97)	0.001*	2.20 (1.51,3.24)	0.001*
Formal education	162	82	1		1	
Number of children						
No children	74	116	1.90 (1.34,2.71)	0.001*	0.98 (0.57,1.66)	0.929
One child	32	48	1.82 (1.12,2.97)	0.016*	1.13 (0.35,1.95)	0.153
Two children	74	84	1.38 (0.95,1.99)	0.090	1.40 (0.48,2.02)	0.234
Three and above child	216	178	1		1	
Source of income						
Retirement	20	14	1		1	
Helped by family	34	114	4.79 (2.19,10.48)	0.001*	3.76 (1.52,8.98)	0.001*
Farmers	170	74	0.62 (0.30,1.30)	0.206	0.60 (0.33,1.12)	0.514
Merchant	34	8	0.34 (0.12,0.94)	0.038*	0.33 (0.48,1.87)	0.065
Helped by NGO	138	216	2.24 (1.09,4.57)	0.028*	1.70 (0.25,2.25)	0.155
Living status						
Alone	144	236	2.17 (1.64,2.88)	0.001*	1.83 (1.35,2.89)	0.006*
With family	252	190	1		1	
Social support						
No	302	248	0.43 (0.32,0.59)	0.001*	0.40 (0.41,1.36)	0.191
Yes	94	178	1		1	
Chronic illness						
No	328	280	1		1	
Yes	68	146	2.52 (1.81,3.50	0.001*	2.21 (1.41,3.40)	0.001*
Current substance use						
No	136	194	1		1	
Yes	260	232	0.63 (0.47,0.83)	0.001*	1.10 (0.61,1.28)	0.880

Poor quality of life was more increased with the advanced age of the elderly. Being singles had two times more likely to have low overall WHOQOL compared to married study participants [AOR = 1.95; 95% CI (1.53, 3.86)]. Regarding Educational status, those with no formal education were two times more likely to have low overall WHOQOL than those with formal education [AOR = 2.20, 95% CI (1.51, 3.24)]. Those living alone were two times more likely to have low overall WHOQOL than their counterparts [AOR = 1.83, 95% CI (1.35, 2.89)]. Regarding income sources, the elderly participants who helped by their families have three times more likely to have a low quality of life than those who have retirement [AOR = 3.76; 95% CI (1.52, 8.98)]. The elderly participants who have chronic diseases were two times more likely to develop low overall WHOQOL when compared with those who have no chronic diseases [AOR = 2.21; 95% CI (1.41, 3.40)] (Table 3).

## Discussion

In this study, we found that the prevalence of depression symptoms and low overall WHOQOL-BREF among the elderly persons was 54.5% and 51.8%, respectively. The prevalence of depression symptoms in our finding is in line with community study done in India (58%) [17], higher than the studies done in Ambo town (41.8%), South Africa (50.1%), South Korean (27.8%), and Pakistan (40.6%) [14, 15, 20, 40]. The prevalence of low quality of life in this study is higher than the study done in India that found 28% of study participants have a poor quality of life [17] and lower than another study reported in India (85.7%) [16]. The difference may be explained by the variation of socioeconomic, cultural factors, sample size, and data collection tools cutoff point.

We found the WHOQOL-BREF sub-domain scores were 49.4% for the physical domain, 43.8% for the psychological domain, 43.8% for the social domain, and 57.7% for the environmental domain of the elderly persons had reported low quality of life. This finding is higher than the finding of the study done in Portugal that found 30% of the physical health domain, 23% of the psychological domain, 19% of the social relationships domain, and 11% of the environment domain [41]. The variation may be due to the lifestyle of elderly persons and the difference between the study design and areas.

In this study, there was a significant association of depression symptoms with increased age. This is similar to studies done in Turkey, Nepal, and Taiwan [42–44]. A study documented that depression is more observed in the elderly aged 70 years and over [45]. Advanced age is also associated with low quality of life. As age increased, the quality of life significantly decreased. This finding is similar to the study done in Turkey [42]. For elderly people, quality of life has decreased at a faster rate as they have gotten older with physical inactivity. It is predicted that as a person ages, the likelihood of physical and health problems including impairment of daily living tasks owing to a lack of disposition or energy, would be increased [46].

Education is an important factor influencing the quality of life and mental health problem. Individuals with a higher educational level had a significantly higher quality of life, on the other hand, individuals with a lower educational level had a low quality of life [47, 48]. We found that elderly persons with no formal education had significantly higher depression symptoms and low quality of life than educated elderly persons. This is similar to the study done in Turkey, which found that no formal education was associated with depression symptoms and poor quality of life [49].

Social support is an imperative factor in sustaining and improving the psychosocial status and well-being of the elderly [50]. In this study, we found that elderly persons with no social support were significantly associated with depression symptoms. Conversely, those elderly who supported by their family had significantly associated with low quality of life. This may be elderly families have no enough to support the elderly due to socioeconomic impact, few family sizes, and poor perception toward the elderly importance. Increased levels of social support have been linked to a lower risk of physical disease, mental illness, and death [51, 52]. Social assistance can be essential for those older individuals who depend on family, friends, or organizations to assist them with everyday tasks, promote companionship, and caring for their quality of life [52].

We found that single elderly participants were significantly associated with the symptoms of depression and low quality of life compared to married elderly participants. Other studies have also reported similar results [53, 54]. Loneliness may play an important psychological role in the development of mental health problems in elderly persons. Prolonged loneliness may jeopardize an individual’s mental well-being and increase the risk of depression, low quality of life, and suicide [55]. We found that living alone was significantly associated with depression symptoms and low quality of life, which was similar to Turkey’s study [42]. Another study also reported depression and quality of life are high in an individual who lives alone [56]. Older adults who almost never visited friends or relatives reported more mentally unhealthy, sad, depressed, worried, anxious, having less energy and low quality of life than those who visited friends or relatives at least several times a week [57].

In this study, we found that elderly persons with chronic disease were significantly associated with symptoms of depression. Different studies have been reported a significant correlation between chronic disease and depression [58, 59]. The elderly who have a chronic disease had a significantly lower quality of life than those without a chronic disease. Similarly, other studies have also found that the presence of a concomitant disease results in a high risk for lower quality of life and psychological distress [54, 60, 61].

The limitation for this study was the participants have questioned mainly events for the symptoms of depression and quality of life that have happened not more than 1 week ago for depression and 2 weeks ago for quality of life to minimize recall bias, but still, there could be. Another limitation of this study may be the cultural factors that impact self-reported symptoms of depression and quality of life. Finally, lack of knowledge about potential pharmacological treatments (medical and psychiatric medicines) or psychological therapy factors linked to depression symptomatology.

## Conclusion

We found the majority of elderly persons have symptoms of depression and low quality of life. Advanced age, living alone, single, lack of formal education, lack of social support, and chronic diseases were significantly associated with the geriatric depression scale. Advanced age, being single, lack of formal education, those elderly who supported by their family, living alone, and having chronic diseases were statistically associated with the overall WHOQOL-BREF low quality of life. Symptoms of depression were correlated with low quality of life. It is imperative that the North Shoa Zone develop psychological interventions, provide ongoing education for the elderly, and monitor the elderly population’s health to address the specific needs of elderly persons who have been impacted by the aging process. In addition, the Ethiopian Ministry of Health should promote the mental well-being and successful aging of Ethiopia’s elderly population.

## Data Availability

The datasets generated and/or analyzed during the current study are not publicly available. Sharing of data was not included in the approval from the ethics committee but is available from the corresponding author on a reasonable request.

## Abbreviations

GBD: Global Burden of Disease
GDS: Geriatric Depression Scale
OWHOQOL-BREF: Overall WHOQOL-BREF
QOL: Quality of life
SLUERC: Salale University ethical review committee
UN: United Nations
WHO: World Health Organization
WHOQOL: WHO Quality of life OLD BREF scale
